# The Potential Role of Zilretta in Reducing Intra-Articular Effusions

**DOI:** 10.31486/toj.25.0027

**Published:** 2025

**Authors:** Kyle Maloney, Taylor Colon

**Affiliations:** ^1^ The University of Queensland Medical School, Ochsner Clinical School, New Orleans, LA; ^2^ Department of Physical Medicine and Rehabilitation, Ochsner Clinic Foundation, New Orleans, LA

**Keywords:** *Osteoarthritis–knee*, *physical and rehabilitation medicine*, *steroids*

## Abstract

**Background:**

Osteoarthritis pain results from articular cartilage degeneration that leads to the breakdown and ulceration of the joint surface accompanied by hypertrophic bone changes with osteophyte formation. Steroids are commonly used to treat osteoarthritis pain.

**Case Report:**

An 81-year-old female with a medical history of bilateral knee osteoarthritis presented to the outpatient physical medicine and rehabilitation clinic for management of chronic bilateral knee pain and effusion. The patient had historically been managed with short-acting steroids, viscosupplementation, and deep genicular radiofrequency ablation. After changing providers, the patient underwent a series of image-guided intra-articular injections with the steroids Kenalog (triamcinolone) and Zilretta (triamcinolone acetonide extended-release injectable suspension) and was also treated with Monovisc (hyaluronic acid viscosupplementation). In addition, the patient underwent cryoneurolysis treatment (iovera° [Pacira BioSciences, Inc]). Injections were administered bilaterally when indicated, while cryoneurolysis was limited to the left knee. A substantial reduction in aspirated effusion volume was observed following Zilretta injections, with 92.6% and 88.1% reductions in aspirate volume in the patient's left and right knees, respectively.

**Conclusion:**

Steroids have anti-inflammatory properties that reduce pain and improve function in patients with osteoarthritis, but steroid use for chronic effusion management also needs to be considered. Given our patient's knee effusion reduction after Zilretta injection, research is needed to confirm the ability of Zilretta to effectively reduce chronic effusion.

## INTRODUCTION

Osteoarthritis is the most common joint disorder in the United States, affecting millions of people each year.^1^ In patients older than 60 years, approximately 10% of men and 13% of women are affected.^2^ Symptomatic pain leads patients to present for management of osteoarthritis.^2^ Pain is the result of articular cartilage degeneration, which leads to the breakdown and ulceration of the joint surface accompanied by hypertrophic bone changes with osteophyte formation.^3^ The gold standard for diagnosis is radiologic investigation.^4^ Narrowed joint space with osteophyte formation is commonly seen in patients with osteoarthritis, and the Kellgren and Lawrence system stratifies the severity with 5 grades: grade 0, no narrowing; grade 1, low chance of articular space constriction, with osteophytic lipping possible; grade 2, moderate chance of articular space constriction with permanent osteophytes; grade 3, definite articular space constriction with end bone deformation and mild osteophytes; grade 4, severe constriction of the joint space, severe sclerosis with bone deformation, and severe osteophytes.^4^

The risk for development of osteoarthritis is multifactorial. Systemic factors (such as genetics, dietary intake, estrogen use, and bone density) and biomechanical factors (such as muscle weakness, obesity, and joint laxity) play a role in the development of osteoarthritis.^1^

Obesity is a concerning risk factor for osteoarthritis,^1^ and since the COVID-19 pandemic, the prevalence of obesity has increased. In 2022, Restrepo documented significantly higher average body mass index (+0.6%, *P*<0.05) and obesity prevalence (+3%, *P*<0.05) rates in the United States during the COVID-19 pandemic compared to the prepandemic period.^5^ Systemic effects of obesity include higher levels of proinflammatory cytokines, adipokines, and matrix metalloproteinases that have destructive effects on articular cartilage and activate synovium.^6^ Given the established link between obesity and the risk for osteoarthritis development, the trend of increasing obesity means that a higher proportion of individuals are at risk for developing osteoarthritis.^1^

One treatment commonly used for osteoarthritis pain is intra-articular steroid injections. Long-acting steroids are commonly used, as they require fewer injections to achieve the same result as short-acting steroids.^7^ Commonly used steroids in the setting of osteoarthritis are methylprednisolone acetate (Depo-Medrol) and triamcinolone acetonide (Kenalog), which can provide relief for 1 to 8 weeks.^8^ Zilretta (triamcinolone acetonide extended-release injectable suspension) is a longer-acting steroid that the US Food and Drug Administration approved in 2017 specifically for osteoarthritis pain of the knee.^9^ However, the disease-modifying effects of Zilretta remain unclear.

## CASE REPORT

An 81-year-old female with a medical history of mitral valve prolapse, hypothyroidism, and headaches presented to the outpatient adult physical medicine and rehabilitation clinic on March 3, 2023, for management of chronic bilateral knee pain and effusion with known bilateral primary knee osteoarthritis. Prior to establishing care at our clinic, the patient was treated by a physical medicine and rehabilitation clinician who managed her osteoarthritis knee pain with short-acting steroids, viscosupplementation, and deep genicular radiofrequency ablation.

Physical examination at presentation showed 5/5 gross motor strength, 2+ reflexes of bilateral lower extremities, full range of motion in flexion and extension of bilateral knees, and tenderness to palpation over bilateral medial and lateral joint lines. X-rays in the patient's medical record from September 2019 revealed bilateral tricompartmental osteoarthritis, Kellgren and Lawrence system grade 2.

Initial management of the patient began with bilateral knee triamcinolone acetonide (Kenalog) 40 mg/mL injections beginning May 15, 2023, followed by bilateral knee hyaluronate sodium 88 mg (Monovisc) injections, cryoneurolysis (iovera° [Pacira BioSciences, Inc]), and eventually extended-release triamcinolone acetonide (Zilretta). Beginning with the patient's appointment in August 2023, management also included aspiration to reduce knee effusion. The aspiration was performed prior to the intra-articular injection and/or procedure.

The Table provides details of the patient's management. Kenalog was administered twice at approximate 3-month intervals. Monovisc was administered twice, with a 5-month duration between treatments. iovera° was performed monthly for 3 consecutive months. Despite the bilateral intra-articular Kenalog injections and iovera° treatments, the patient endorsed unchanging knee pain. Further, the 2 Kenalog injections had little effect on the volume of aspirate from the patient's knees.

**Table.** Osteoarthritis Management, August 2023 through June 2024

	Aspiration Volume, cc		Percentage Change From Previous Appointment		
Date	Left Knee	Right Knee	Left Knee	Right Knee	Injections/Approvals/Procedures
August 10, 2023	16.5	37	–	–	Bilateral intra-articular Kenalog injections
September 12, 2023	–	–	–	–	Insurance approval for iovera° (Pacira BioSciences, Inc) submitted
October 26, 2023	–	–	–	–	Approved for iovera° Because of greater pain in left knee, left superficial genicular nerves treated with iovera°
November 20, 2023	17	14	+3.0	–62.2	Unchanged left knee pain Bilateral intra-articular knee Kenalog injections iovera° treatment in the left knee
December 19, 2023	27	41	+58.8	+192.9	Bilateral intra-articular knee Monovisc injections iovera° treatment in the left knee
March 19, 2024	27	42	+0.0	+2.4	Bilateral intra-articular knee Zilretta injections
May 23, 2024	2	5	–92.6	–88.1	Bilateral intra-articular Monovisc injections
June 19, 2024	0	0	–100	–100	Bilateral intra-articular Zilretta injections

Note: All aspiration and injection procedures were performed under ultrasound guidance.

After the patient was transitioned to Zilretta injections on March 19, 2024, the volume of knee aspirate dropped approximately 90% in both knees on May 23, 2024, and then decreased to zero at the following appointment on June 19, 2024. The Figure depicts the aspiration volume at each appointment from August 2023 through June 2024.

**Figure.**
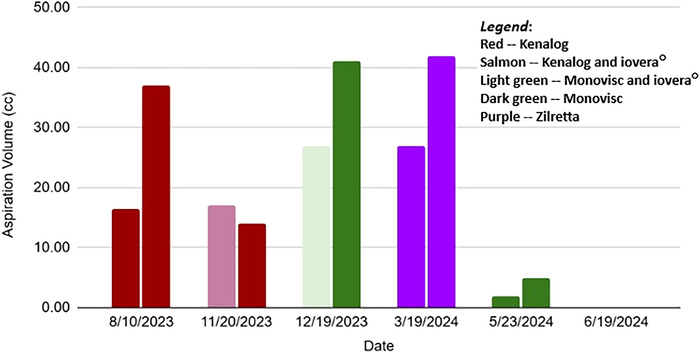
Aspiration volume by appointment date and treatment received. For each appointment date, the left bar of the graph corresponds to the left knee, and the right bar corresponds to the right knee.

Following the June 2024 data collection endpoint for this case report, the patient was continued on Zilretta and Monovisc injections and iovera° treatments.

## DISCUSSION

To our knowledge, no literature examining the role of Zilretta in intra-articular effusion management exists. Available data about Zilretta primarily assess pain outcomes and duration of pain relief relative to placebo and triamcinolone acetonide crystalline suspension, with no focus on the volume of aspirate.^10^ Our patient's experience suggests that Zilretta may have benefits beyond analgesia; the extended-delivery capsule may allow for more sustained anti-inflammatory effects in the intra-articular microenvironment.

Clinically, this finding could be applied to other patients with recurrent effusions in the setting of osteoarthritis. However, the applicability of these findings to patients with similar presentations is unknown. Consequently, research studies are needed to assess the relationship between Zilretta use and reduced intra-articular effusion volume.

## CONCLUSION

This case report suggests that in addition to reducing pain and improving function in patients with osteoarthritis, Zilretta may play a role in reducing chronic knee effusion. Studies exploring the effects of Zilretta on aspirate volume are needed.

## Notes

*This article meets the Accreditation Council for Graduate Medical Education and the American Board of Medical Specialties Maintenance of Certification competencies for Patient Care and Medical Knowledge.*
